# Deleterious Effects of Chronic Folate Deficiency in the Ts65Dn Mouse Model of Down Syndrome

**DOI:** 10.3389/fncel.2017.00161

**Published:** 2017-06-09

**Authors:** Susan Helm, Morgan Blayney, Taylor Whited, Mahjabin Noroozi, Sen Lin, Semira Kern, David Green, Ahmad Salehi

**Affiliations:** ^1^Natural Science Division, Pepperdine UniversityMalibu, CA, United States; ^2^VA Palo Alto Health Care System (VAPAHCS)Palo Alto, CA, United States; ^3^Department of Psychiatry and Behavioral Sciences, Stanford University School of MedicinePalo Alto, CA, United States

**Keywords:** Down syndrome, folic acid, hippocampus, homocysteine, methylation, Ts65Dn mice

## Abstract

Folate is an important B vitamin naturally found in the human diet and plays a critical role in methylation of nucleic acids. Indeed, abnormalities in this major epigenetic mechanism play a pivotal role in the pathogenesis of cognitive deficit and intellectual disability in humans. The most common cause of cognitive dysfunction in children is Down syndrome (DS). Since folate deficiency is very common among the pediatric population, we questioned whether chronic folate deficiency (CFD) exacerbates cognitive dysfunction in a mouse model of DS. To test this, adult Ts65Dn mice and their disomic littermates were chronically fed a diet free of folic acid while preventing endogenous production of folate in the digestive tract for a period of 8 weeks. Our results show that the Ts65Dn mouse model of DS was significantly more vulnerable to CFD in terms of plasma homocysteine and N5-methyltetrahydrofolate (5-MTHF) levels. Importantly, these changes were linked to degenerative alterations in hippocampal dendritic morphology and impaired nest building behavior in Ts65Dn mice. Based on our results, a rigorous examination of folate intake and its metabolism in individuals with DS is warranted.

## Introduction

Folates comprise the essential B9 vitamin that, for mammals, must be obtained from the diet. While folates are entirely derived from the diet, folic acid is the more stable form of folate and is used in food fortification. Folates are needed for one-carbon metabolism in both biosynthesis and methylation of RNA and DNA precursors. The process of DNA methylation is considered a major epigenetic phenomenon and plays a significant role in neuronal functions (Hamdane et al., 2016), including memory processing. For these reasons, abnormalities in folate intake or its metabolism could potentially lead to learning disabilities. It has been shown that mutations in 5-methyltetrahydrofolate-homocysteine methyltransferase reductase (MTRR) gene in humans led to developmental delay, brain atrophy, seizures, hypotonia and dementia. Importantly, all these abnormalities have also been reported in people with Down syndrome (DS; Arumugam et al., 2016). For instance, reducing the expression of folate transporters in mice leads to a significant failure in spatial learning (Höger et al., 2009). Furthermore, mutations in the methylenetetrahydrofolate reductase (MTHFR) gene or those with deficiency in MTRR show hyperhomocysteinemia, failure in DNA methylation (Rozen, 2000), and novel object recognition impairment (Jadavji et al., 2014). Due to the essential role of folate metabolism in cognitive function and the fact that a considerable proportion of the pediatric population is exposed to suboptimal doses of folate intake, the question that was raised was whether chronic folate deficiency (CFD) could lead to exacerbation of symptoms in individuals with cognitive disability.

There are at least 200,000 individuals with DS in the US suffering from life-long deficits in cognitive function and abnormalities in multiple organs (de Graaf et al., 2015). DS is primarily caused by chromosomal nondisjunction and thus overexpression of ~500 genes mapped to human chromosome 21 (Hsa21). The affected individuals suffer from lifelong cognitive disability along with numerous abnormalities in the cardiovascular, hematopoietic and digestive systems (McNerney et al., 2017). The molecular mechanisms of cognitive deficit in DS are yet to be fully understood. The synteny between Hsa21 and mouse chromosome 16 (Mmu16) has been an important factor in generating mouse models of DS and elucidating the pathogenesis of cognitive dysfunction in DS. From this group, the Ts65Dn mouse with a triplication a fragment of Mmu16 is the most well characterized. While a number of successful preclinical studies have been performed in the Ts65Dn mouse model (Salehi et al., 2009; Dang et al., 2014; Kazim et al., 2017), an effective therapeutic strategy is yet to be developed to improve cognitive function in people with DS. Indeed, identifying genetic, biochemical, and environmental factors that can moderate cognitive dysfunction in DS could play an important role in improving the quality of life in people with DS (Millan Sanchez et al., 2012).

Chromosome 21 was the first human chromosome to be fully sequenced (Hattori et al., 2001). Careful genetic mapping along with clinical studies during the last several years have attracted attention to a strong relationship between folate metabolism and DS: (i) out of >500 Hsa21 genes triplicated in DS, several genes including reduced folate carrier (RFC1 or SLC19A1) and Cystathionine β-synthase (CBS) have been linked to folate metabolism (Gardiner, 2015); (ii) Hsa21 has a high frequency of regions with altered methylation; and (iii) failure in folate metabolism leads to learning disability in humans. These data support the notion that folate levels can strongly moderate cognitive dysfunction phenotype in DS. A large number of studies have established a relationship between increased levels of plasma homocysteine (Hcy) and abnormal DNA methylation in several human disorders (Ingrosso et al., 2003; Bönsch et al., 2004; Huang et al., 2009; Lv et al., 2011). For this reason, we quantified plasma levels of this amino acid in mice undergoing FA deficiency as an indication of DNA methylation.

A number of DS functional and structural phenotypes have been faithfully recapitulated in animal models overexpressing genes triplicated in DS (Salehi et al., 2007). During the last several years, we have studied the Ts65Dn mouse model of DS. These mice have triplication of a segment of Mmu16 orthologous to that of Hsa21. Extensive behavioral and morphometrical studies have indicated that adult Ts65Dn mice show significant failure in structure and function of the hippocampal region (Salehi et al., 2009; Mojabi et al., 2016). Here we assessed the effects of CFD and asked whether it could further exacerbate phenotypes caused by overexpression of mouse genes orthologous to those mapped to Hsa21.

As we report here, the Ts65Dn mouse model of DS showed significant increase in plasma Hcy and folate metabolite levels N5-methyltetrahydrofolate (5-MTHF) following dietary CFD. These changes were associated with failure in hippocampal-mediated behavior and degenerative alterations in dendritic tree in the hippocampal region in Ts65Dn mice.

## Materials and Methods

### Mice

Four-month-old male Ts65Dn (B6EiC3Sn.BLiA-Ts(17<16>)65Dn/DnJ) (Stock #005252) and their disomic (2N) littermates were purchased from the Jackson Laboratory (Bar Harbor, ME, USA) and housed at the Pepperdine University Animal Facility. All animal experiments were performed within the guidelines of the National Institutes of Health, Guide for the Care and Use of Laboratory Animals and approved by Institutional Animal Care and Use Committee (IACUC) of Pepperdine University. The mice were maintained as groups of four throughout the study. Mice were housed under a controlled environment (12 h light/dark cycle, 18–24°C) with *ad libitum* access to food and water. Following 5 weeks of habituation, the 2N and Ts65Dn mice were randomly divided into two groups. The first group was fed with an experimental diet from Harlan Teklad^®^, devoid of folic acid. To inhibit endogenous folate production by intestinal flora, the diet contained 2.5 g/kg choline bitartrate and 3.0 g/kg L-cystine. The second group received pellets containing 2 mg/kg folic acid, the Recommended Dietary Allowance for rodents set by the American Institute of Nutrition.

### Behavioral Assessment

Nesting was used as a measure of cognitive ability and attention. It has been shown that nesting is a hippocampal-mediated cognitive function (Deacon et al., 2002) and could be used as a reliable measure of attention in Ts65Dn mice (Antonawich et al., 1997; Heller et al., 2014; Olmos-Serrano et al., 2016). In fact, in a recent study (Heller et al., 2014) nesting alone was used as a reliable marker for attention in the Ts65Dn mouse model of DS. To conduct this measure, the ability of the mice to build a nest from a nestlet (Nestlet™, Ancare, NES3600) was quantified. Sixty minutes prior to the dark period, each mouse was placed into an individual, unfamiliar environment (rectangular cage) with a nestlet, then allowed 12 h to complete the test. The cage was lined with fresh bedding, *ad libitum* experimental/control diets and fresh water. A pre-weighed single nestlet was placed in each cage. Overnight, each individual mouse had unrestricted access to the nestlet, then, after 12 h, the condition of each nestlet was observed, scored and photographed. The amount of unused nestlet was weighed. The nesting score was calculated as the percentage of the nestlet used by each mouse. Once nesting activity assessment was completed, all mice were returned to their corresponding home cages.

### Analysis of Plasma Homocysteine Levels

The day after completion of the nesting experiment, all mice were sacrificed and blood and brain were collected for further analysis. The mice were deeply anesthetized using Nembutal^®^ sodium solution CII (Oaks Pharmaceuticals, Inc., Lake Forest, IL, USA, NDC: 76478-501-20). Blood was collected by cardiac puncture in potassium-EDTA coated tubes and plasma was separated and stored at −80°C. The vials containing 50 mM phosphate buffer and 10 mM EDTA (pH 7.4) and plasma were centrifuged at 14,000 *g* for 10 min. For derivatization, a 50 μL aliquot of plasma was mixed with 10 μL of tri-n-butylphosphine (100 μL/mL in DMF), and incubated at 4°C for 30 min. Proteins were then precipitated with the addition of 50 μL of 10% trichloroacetic acid and incubating at room temperature for 10 min. The samples were centrifuged for 10 min at 16,000 *g*. A 50 μL aliquot of the supernatant was transferred to a vial containing 10 μL of 1.55 M NaOH, 200 μL of 125 mM borate buffer with 4 mM EDTA (pH: 9.5), and 50 μL of SBD-F (ammonium 7-fluorobenzo-2-oxa-1,3-diazole-4-sulfonate, Fisher Scientific, Waltham, MA, USA) solution (1.0 g/L in 125 mM borate buffer, pH: 9.5). The samples were incubated at 60°C for 1 h. Standard samples of authentic Hcy were treated identically. Hcy was determined by high-performance liquid chromatography adapted from the literature (Minniti et al., 1998). Analysis of samples was performed by reversed-phase HPLC using a Luna C18 column (150 × 4.6 mm, 5 μm *d*p, Phenomenex, Inc., Torrance, CA, USA) protected with a guard cartridge (SecureGuard^®^, Phenomenex) coupled to a Thermo Separations P4000 pump and equipped with a Waters 2475 multi-wavelength fluorescence detector with an excitation wavelength of 385 nm and an emission wavelength of 515 nm. Isocratic separation was performed using 92% 50 mM phosphate buffer (pH: 2.1) and 8% acetonitrile at a flow rate of 1 mL/min. The manual injection volume was 20 μL and samples were filtered through a 2 mm diameter 0.2 μm porosity PVDF membrane syringe-tip filter (Millipore, Billerica, MA, USA) during the injector load step. A rectilinear calibration curve was prepared for Hcy.

### 5-Methyltetrahydrofolate Levels

**Figure 1:**
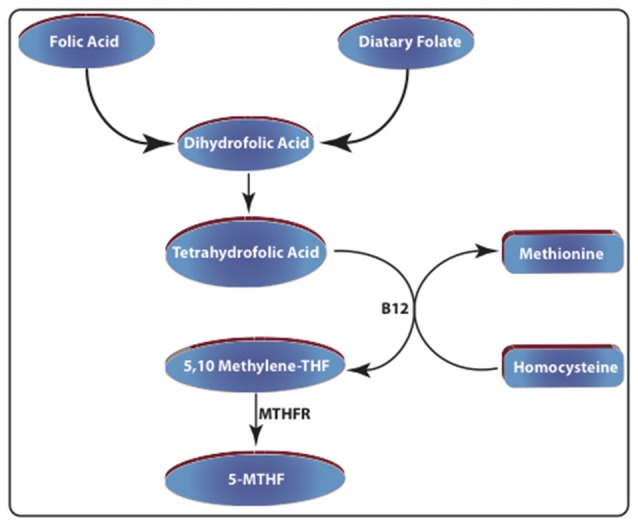
Schematic representation of folate metabolism. Dietary folate and folic acid-enriched foods are considered the major sources of dihydrofolic acid. This compound is reduced to tetrahydrofolic acid (THF) by dihydrofolate reductase (DHFR). Then it is converted into 5,10-methylene tetrahydrofolic acid by serine hydroxymethyltransferase (SHMT). The enzyme methylenetetrahydrofolate reductase (MTHFR), converts this compound into N5-methyltetrahydrofolate (5-MTHF). Increased folate levels also promote the conversion of homocysteine to methionine by methionine synthase (MS).

We quantified the levels of 5-MTHF, an end product of folate metabolism in plasma. MTHFR is a flavoprotein that catalyzes the NADPH-dependent conversion of 5,10-methyltetrahydrofolate (5,10-MTHF) to 5-MTHF (Figure 1). For 5-MTHF quantification in plasma, an enzyme-linked immunosorbent assay (ELISA) was employed with an antibody specific for 5-MTHF (CEG81Ge, Cloud-Clone Corp., Katy, TX, USA). A competitive inhibition reaction was launched between horseradish peroxidase-labeled-5-MTHF and unlabeled 5-MTHF. After the incubation of plasma samples in a pre-coated plate, the unbound conjugate was washed off. Following the addition of tetramethylbenzidine (TMB), the samples were quantified spectrophotometrically (SpectraMax^®^ Plus 384 Microplate Reader, Molecular Devices, Sunnyvale, CA, USA).

### Brain Extraction and Golgi Staining

Following deep anesthesia, the brain of each mouse was removed and immediately placed in a glass tube. The brains were immediately immersed in 15 mL of undiluted Golgi solution and stored in the dark at room temperature (Das et al., 2015). The solution was replaced on the second day and returned to the dark at room temperature for an additional 9 days. Finally, the brains were incubated for 3 days at 4°C in 30% sucrose solution (Sigma-Aldrich) for dehydration. Each brain was firmly mounted on the vibratome platform using superglue and sliced into 150 μm thick sections at room temperature. Sections were put on gelatinized slides and coated with 30% sucrose and air-dried in a dark room for 3 days. The slides were immersed in a diluted developing solution for 7–10 min and rinsed in double distilled H_2_O. Water was removed by soaking the slides in increasingly higher concentrations of ethanol(s) for 10 min each. The slides were coverslipped using di-n-butyl phthalate in xylene (Sigma-Aldrich) as mounting medium and kept in 100% xylene (for detailed protocol see Das et al., 2015).

### Dendritic Arborization Assessment

We used a high-throughput digital microscope (Keyence BZ-X710) for image acquisition. Slides were loaded to the digital microscope cassette and the images were automatically collected from the dentate gyrus using a 40× objective (PlanApo40x, 0.95/0.25-0.16 mm). The size and quantity of the basal dendrites were determined with ImageJ (Version 2.0.0-rc-54/1.51h) program. Following calibration, the minimum distance between the cell body border and first dendritic tree bifurcation was manually measured in the apical dendrites of dentate granule neurons.

### Statistical Methods

All results are expressed as mean ± SEM. Due to the lack of normal distribution of the obtained data, a non-parametric Kruskall-Wallis test followed by the Mann-Whitney U test were used for comparisons. The results of statistical comparisons were considered significant at *p* < 0.05.

## Results

Each mouse was fed with folate-deficient- or control pellets for a period of 8 weeks in their home cages. The mice were observed and weighed weekly for signs of distress or severe side effects. While the Ts65Dn group had significantly lower body weight compared to 2N mice (*p* < 0.001), no significant decline in body weight was detected during the study (Supplementary Figure S1). At the end of the 8-week treatment, all mice were assessed for the nesting behavior and sacrificed a day after, for biochemical and morphometric studies.

### Nesting Behavior

**Figure 2:**
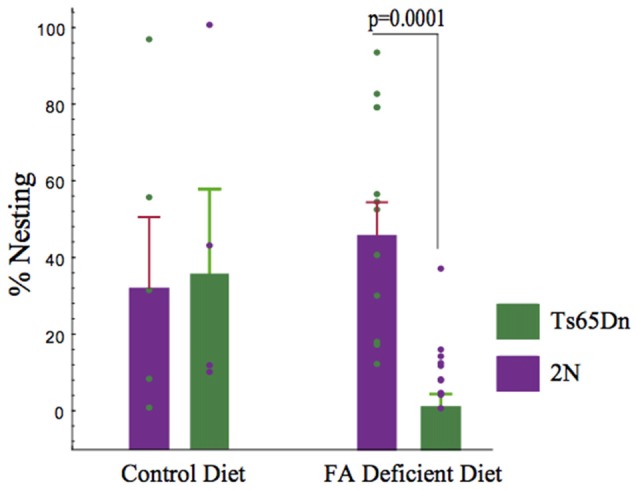
The effects of chronic folate deficiency (CFD) on the nesting behavior in adult Ts65Dn and their 2N controls. A significant decrease in nesting was observed in Ts65Dn mice. Quantification of nesting (amount of nestlet used) showed a deficiency of folate in Ts65Dn mice is more harmful to nesting score than the 2N group. A non-parametric Kruskall-Wallis test was used to test the overall differences among all four groups and showed a significant difference (*p* = 0.005, H (3, *n* = 34)). We found no significant deficiency in nesting in mice receiving normal levels of folate (2N = 37.923 ± 17.443, *n* = 5, Ts65Dn = 41.239 ± 20.904, *n* = 4, *p* > 0.05). Importantly, inducing CFD led to a significant (~5 fold) reduction in hippocampal-mediated nesting behavior in Ts65Dn mice (2N-CFD = 50.767 ± 8.127, *n* = 12, Ts65Dn-CFD =10.509 ± 2.988, *n* = 11, *p* = 0.0001).

Nesting is a hippocampal-mediated behavior. Laboratory rodents build nests for a variety reasons, including thermoregulation. Quantification of nesting (amount of nestlet used) in 2N and Ts65Dn-CFD mice showed a decline in nesting scores in Ts65Dn-CFD mice. Non-parametric Kruskall-Wallis test showed significant effects of treatment and genotype in both 2N and Ts65Dn groups (*p* = 0.006, H (3, *n* = 31)). While we found no significant impairment in the nesting behavior in 2N mice (*p* = 0.624), CFD led to a significant (~5 fold) reduction in hippocampal-mediated nesting behavior in Ts65Dn mice (Figure 2).

### Hcy Levels in Plasma

**Figure 3:**
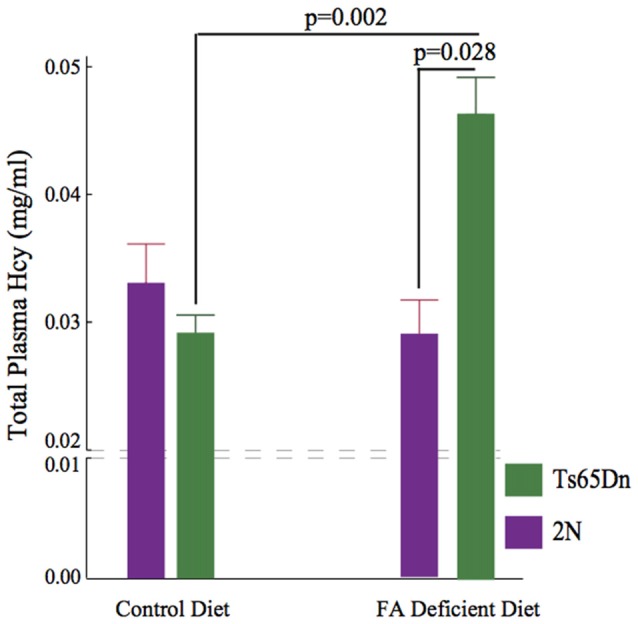
Elevation of plasma Homocysteine (Hcy) levels following CFD. Kruskall Wallis test showed a significant difference among four groups (*p* = 0.011, H (3, *n* = 32)). No difference was found between 2N-CFD and 2N (2N = 0.291 ± 0.029, *n* = 11, 2N-CFD = 0.332 ± 0.032, *n* = 5). However, there was a significant increase (~50%) in Ts65Dn-CFD compared with Ts65Dn mice fed control diet (Ts65Dn = 0.293 ± 0.012, *n* = 11, Ts65Dn-CFD = 0.458 ± 0.038, *n* = 5, *p* = 0.002). As the result, there was a significant difference between 2N-CFD and Ts65Dn-CFD (*p* = 0.028).

Hcy is converted into methionine during folate metabolism. We quantified total Hcy levels in plasma samples using HPLC. The non-parametric Kruskall-Wallis test showed a significant difference among the four groups (*p* = 0.011, H (3, *n* = 32)). Importantly, while no significant effects of CFD deficiency were found in 2N mice, a significant increase (~50%) was found in Hcy levels following CFD in Ts65Dn mice (*p* = 0.002, Figure 3).

### Plasma 5-MTHF Levels

**Figure 4:**
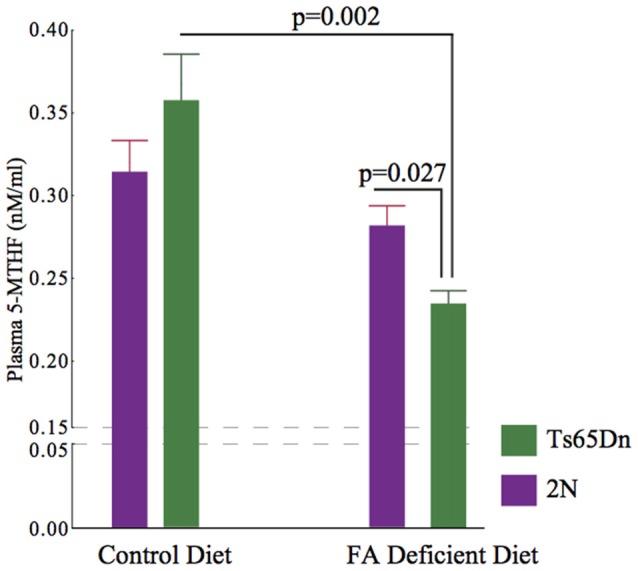
Plasma levels of 5-MTHF in 2N and Ts65Dn mice. A significant difference was found among all four groups (*p* = 0.0029), H (3, *n* = 31). While there was no significant effect in 2N mice (2N-CFD = 0.280 ± 0.0129, *n* = 5, 2N = 0.316 ± 0.017, *n* = 11, *p* = 0.307), there was a significant reduction in Ts65Dn mice (Ts65Dn-CFD = 0.231 ± 0.077, *n* = 5, Ts65Dn = 0.0359 ± 0.025, *n* = 10, *p* = 0.002). As the result, there was a significant difference between 2N-CFD and Ts65Dn-CFD (*p* = 0.027).

The folate metabolism pathway leads to the synthesis of 5-MTHF using the enzyme MTHFR. For this reason, we quantified the plasma levels of 5-MTHF using an ELISA kit. We found significant effects of genotype and treatment on plasma 5-MTHF levels (*p* = 0.0029), H (3, *n* = 31)). While there were no significant effects of CFD in 2N mice (*p* = 0.307), there was a significant (~54%) reduction in 5-MTHF levels in Ts65Dn mice undergoing CFD (*p* = 0.002, Figure 4).

### Hippocampal Dendritic Analysis

**Figure 5:**
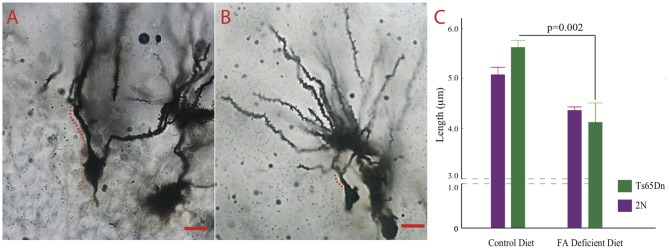
The effects of CFD on dendritic trees in the hippocampus.** (A,B)** Micrographs depicting Golgi-stained dentate granule neurons in the dentate gyrus of a Ts65Dn treated with normal and another one with folate deficient diet. The distance between the cell body and the first bifurcation is labeled. Scale bar = 10 μm. **(C)** The result of quantification of the length between the cell body and the first bifurcation in apical dendrites. While we found relatively no effects of folate deficiency in 2N mice (2N-CFD = 0.884 ± 0.024, *n* = 3, 2N = 0.013 ± 0.0360. *n* = 9, *p* = 0.052), we found a significant reduction in dendritic length in Ts65Dn mice undergoing CFD (Ts65Dn-CFD = 0.805 ± 0.082, *n* = 3, Ts65Dn = 1.121 ± 0.032, *n* = 6, *p* = 0.020). Kruskall Wallis test showed a significant difference among the four groups (*p* = 0.0102, H (3, *n* = 21)).

High levels of an important folate transporter (solute carrier family 19, member 1, SLC19A1) can be found in axons and dendrites (Wang et al., 2001). Furthermore, Ts65Dn mice show significant alterations in dendritic morphology (Dang et al., 2014; Hannula and Duff, 2017). We tested the effects of CFD on dendrites in the dentate gyrus of Ts65Dn mice. These cells play a significant role in attention and contextual learning (Dang et al., 2014). The Kruskall-Wallis test showed a significant difference among the four groups tested (*p* = 0.0102, H (3, *n* = 21)). While we found no statistically significant effects of CFD in 2N mice (*p* = 0.052), we found a significant reduction in dendritic length in Ts65Dn mice undergoing CFD (*p* = 0.020; Figures 5A,B).

## Discussion

While most attention in DS has been paid to the role of folate deficiency in the etiology of chromosomal non-disjunction and the beneficial effects of folate supplementation (Coppedè, 2016), the possible detrimental effects of CFD in children and adults with DS have been largely overlooked. Here we found that CFD in Ts65Dn mice could produce more drastic metabolic, functional, and structural alterations in the Ts65Dn mouse model of DS compared with normozomic (2N) mice.

Folate is a water-soluble vitamin and is considered a 1-carbon source in several critical cellular pathways including DNA, RNA, and protein methylation (Hamdane et al., 2016). Indeed, folate is required for remethylation of Hcy to methionine (Figure 1). DNA methylation is one of the primary epigenetic mechanisms in both rodents and humans and plays a critical role in maintaining the genome and regulating gene expression (Perri et al., 2017). The decline of overall DNA methylation has been associated with neuronal aging and linked to cognitive dysfunction in DS (Toma et al., 2016). For instance, deleting DNA methyltransferase (Dnmt1 and 3a) in mice leads to cognitive deficits (Griñán-Ferré et al., 2016). Moreover, mutations in Dnmt1 gene in humans have been linked to neurodegenerative alterations (Pedroso et al., 2013).

DS is primarily caused by Hsa21 non-disjunction. Importantly, deep methylation analysis has revealed a high frequency of differentially-methylated regions (DMRs) in Hsa21 (Bacalini et al., 2015). For this reason, individuals with DS are predictably more vulnerable to methylation disorders. As mentioned above, methylation plays an important role in DNA synthesis (Hamdane et al., 2016). Alterations in methylation and failure of DNA synthesis in DS could be linked to a number of DS clinical manifestations. For instance, macrocytosis or increased mean corpuscular volume of erythrocytes in the absence of anemia is common in people with DS (Wachtel and Pueschel, 1991). The increased size of erythrocytes is commonly associated with increased concentrations of hemoglobin (Muravlyova et al., 2015). It has been suggested that folate deficiency is an important causative factor in macrocytosis (Gilfix, 2014). Indeed, reduced folate levels lead to failure in DNA synthesis while sparing RNA synthesis, leading to disproportionate erythrocyte shape (see Imaga, 2013).

There are several Hsa21 genes triplicated in DS that encode proteins involved in folate metabolism. From this group, overexpression of genes like CBS, increases folate demand in people with DS. CBS participates in Hcy metabolism and converts Hcy into cystathionine (Figure 1). Triplication of CBS gene leads to increased levels of CBS protein in the cortex of both fetuses and adults with DS (Ichinohe et al., 2005). Furthermore, ~50% reduction in plasma concentrations of methionine has been reported in DS (Pogribna et al., 2001). Mutations in the CBS gene lead to homocystinuria characterized by elevated plasma Hcy and cognitive durability (El Bashir et al., 2015). RFC1 is another Hsa21 gene that encodes a major folate transporter that uptakes folate from the blood. Through either increased production of reactive oxygen species (ROS) or weakening the cellular antioxidant defense, Hcy can lead to many abnormalities including lipid peroxidation and DNA damage. For this reason, effective cellular mechanisms are needed to detoxify or remove Hcy from the body, commonly achieved by either transsulfuration or remethylation.

A number of preclinical and clinical studies support the role of folate in cognitive function. For instance, it has been shown that individuals with mild cognitive impairment respond positively to folate intake. Dietary folate equivalent levels per day were positively correlated with the word list recognition and memory scores in these individuals (Kim et al., 2014). In addition, several longitudinal clinical studies have suggested a positive correlation between homocysteine levels and the risk of Alzheimer’s disease.

Nesting is common and complex behavior in rodents. The main motivations behind non-reproductive nesting include heat conservation and sheltering from competitors. Nest building is also associated with increased lifetime of reproductive success (Bult and Lynch, 1997). A number of studies have shown that is a hippocampal-mediated function in rodents. Indeed, hippocampal lesions lead to failure in nesting behavior in mice (Deacon et al., 2002; Heller et al., 2014). We have found that targeting 5-HT-receptors using a 5-HT2a blocker and improving brain norepinephrine levels both lead to a significant improvement in nesting behavior in adult Ts65Dn mice (Salehi et al., 2009; Heller et al., 2014). While due to long-term nature of the current experiment, requiring numerous body weight measurements, we failed to detect a significant difference between adult 2N and Ts65Dn mice in the nesting behavior, CFD led to a significant reduction in this behavior in Ts65Dn mice without significantly altering the 2N group (Figure 2). The reason for the differential response to CFD between Ts65Dn and 2N mice may be due to underlying weak compensatory mechanisms related to overexpression of triplicated Hsa21 genes. Uracil-DNA N-glycosylase (UNG) is a glycosylase that removes uracil from DNA (Kavli et al., 2002). It has been shown that Ung null mice undergoing CFD show a significant failure in hippocampal-mediated spatial learning (Kronenberg et al., 2008). An important limitation to the interpretation of the nesting experiment in this study is employing a relatively low number of mice. While despite this low number, we could detect significant effects of folate deficiency in Ts65Dn mice, the nesting experiment must be performed in a larger group of mice in future studies for further verification.

We found that CFD led to a significant increase in plasma Hcy levels in Ts65Dn mice (Figure 3). Hcy is a non-protein forming, non-DNA-coded, and thiol-amino acid that is recycled into methionine or converted into cysteine with the aid of folate and vitamins B6 and B12. For this reason, plasma Hcy concentrations are inversely correlated with folate levels. Hcy is generally toxic and increases the production of ROS (Tsen et al., 2003; Ansari et al., 2014). Several human conditions associated with increased levels of Hcy, i.e., homocystinuria, are associated with cognitive disability, seizures and numerous musculoskeletal and vascular abnormalities. Hcy levels are considered strong predictors of performance in neuropsychiatric symptoms (Hultberg et al., 2001). We found that unlike in 2N mice, CFD in Ts65Dn mice led to a significant increase in Hcy plasma levels. The exacerbated response to CFD in Ts65Dn mice could be due to overexpression of a number of Mmu16 triplicated genes causing increased vulnerability to folate mis-metabolism. Our results are similar to those of De la Torre et al. (2014) detecting no significant differences in basal levels of plasma Hcy between 2N and Ts65Dn. A number of studies have suggested the role of folate metabolism abnormalities in depression (Reynolds, 2002). As we showed here, the induction of CFD in Ts65Dn mice led to increased levels of Hcy in Ts65Dn mice (Figure 3) along with decreased plasma levels of 5-MTHF (Figure 4) in these mice. Hcy is produced from methionine primarily through remethylation by methionine synthase (MS) which, also converts 5-MTHF into tetrahydrofolate (THF) and can be recycled back to 5-MTHF (Figure 1). It has been shown that adjunct therapy with anti-depressants and 5-MTHF is more effective in reducing the depressive symptoms than monotherapy (Passeri et al., 1993). For this reason, we can speculate that the long-term effects of folate deficiency in individuals with DS might lead to exacerbation of depressive symptoms in these individuals (Walker et al., 2011).

Lower folate concentrations have been associated with poorer spatial copying skills in men aged 54–81 years (Riggs et al., 1996). Furthermore, individuals with low folic acid levels display impairment in both word- and object-recall (Eussen et al., 2002).

We also studied the effects of CFD on dendritic structure in 2N and Ts65Dn mice (Figure 5). The extent of dendritic arborization has been shown to be a reliable marker for neuronal degeneration (Dang et al., 2014; Das et al., 2015). We found that CFD led to a significant reduction in dendritic length in dentate granule neurons. These neurons play a significant role in cognitive function. It has recently been shown that inducing pseudo MTHFR deficiency in pregnant mice through high dietary doses of folic acid leads to atrophy of the dentate gyrus and failure in cognitive function in the offspring (Bahous et al., 2017).

It must be noted that while we did not find differences in folate metabolism between naive 2N and Ts65Dn mice, we were able to detect a number of abnormalities in Ts65Dn mice following 8 weeks of CFD. This suggests that folate deficiency in Ts65Dn can potentially unearth a series of underlying abnormalities in the structure and function of hippocampal region in these mice.

## Clinical Implications

The fact that no strong and consistent positive effects of folic acid supplementation have been reported in humans suggests that an effective preventative strategy is more effective than supplementation. A system for detection and prevention of folate deficiency in children and adults with DS might be an effective strategy to prevent further deterioration of functional and structural abnormalities in DS. Since folate deficiency is one of the most common vitamin deficiencies in adolescents (Bailey et al., 1982), careful quantification and surveillance of folate levels in children particularly those with trisomy Hsa21 might be the most effective and economically sound strategy. This will potentially prevent dysregulation in folate metabolism and structural and functional consequences in the nervous system. The other source of concern for DS population is the occurrence genetic variations that increase the risk of CFD. Polymorphism and substitutions in genes encoding the folate-metabolizing enzyme, MTHFR, can alter Hcy levels. Two common substitutions in MTHFR include Ala222Val and Glu429Ala. Between these two, Ala222Val has been linked to increased plasma Hcy and decreased serum folate levels (Ueland et al., 2001). Accordingly, DS individuals with MTRR2756AG substitution, show increased plasma Hcy levels (Biselli et al., 2008). For these reasons, it is imperative to screen DS children for folate deficiency and elevated plasma Hcy levels particularly in those with MTRR2756AG polymorphism.

## Author Contributions

SH: initiated the project and supervised all experiments on a daily basis. MB: performed all sample collections. TW: performed all behavioral testings and sample collection. MN: performed all morphometrical analyses on Golgi-stained samples. SL: performed all ELISA qualifications. SK: performed all sample collections and tissue processing. DG: performed HPLC quantifications of Hcy levels in plasma. AS along with SH, designed the experiments and primarily wrote the manuscript.

## Conflict of Interest Statement

The authors declare that the research was conducted in the absence of any commercial or financial relationships that could be construed as a potential conflict of interest.

## Supplementary Material

FIGURE S1 The effects of folate deficiency on the average body weight (±SEM) of 2N and Ts65Dn mice during the study. While Ts65Dn mice had significantly lower body weight than the 2N group (*p* < 0.001), no significant effects of folate deficiency on body weight were detected in either Ts65Dn or 2N mice (*p* > 0.05).

## Abbreviations

5-MTHF: N5-methyltetrahydrofolate
5,10-MTHF: N5,N10-methyltetrahydrofolate
CBS: Cystathionine β-synthase
CFD: Chronic folate deficiency
DHFR: Dihydrofolate reductase
DMRs: Differentially methylated regions
Dnmt: DNA methyltransferase
DS: Down syndrome
Hcy: Homocysteine
Hsa21: Human chromosome 21
Mmu16: mouse chromosome 16
MS: Methionine synthase
MTHFR: Methylenetetrahydrofolate reductase
MTRR: Methyltransferase reductase
RFC1: Reduced folate carrier
SHMT: Serine hydroxymethyltransferase
SLC19A1: Solute carrier family 19 member 1
THF: Tetrahydrofolate
TMB: Tetramethylbenzidine
Ts65Dn: Trisomy DS mouse model
UNG: Uracil-DNA N-glycosylase.
