# Supplementary figures and images for: Deleterious Effects of Chronic Folate Deficiency in the Ts65Dn Mouse Model of Down Syndrome
Source: Front Cell Neurosci. 2017 Jun 9;11:161. doi: 10.3389/fncel.2017.00161 (PMC5465284; doi:10.3389/fncel.2017.00161)

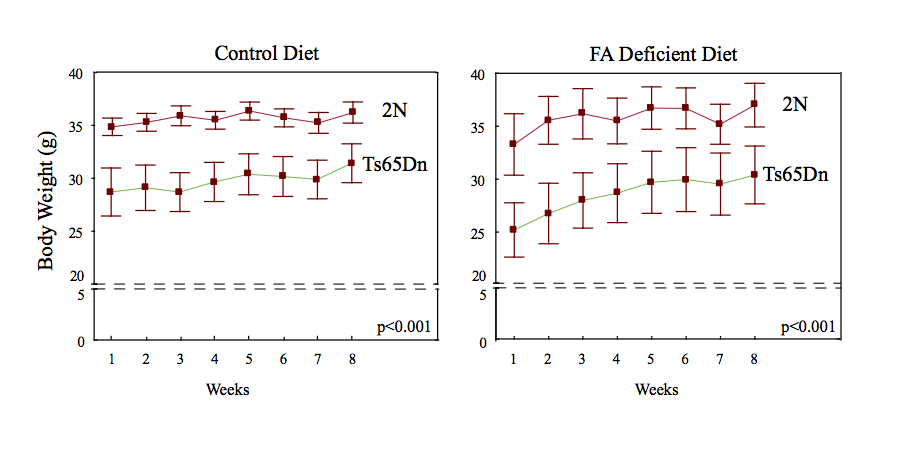

Supplement: FIGURE S1 — The effects of folate deficiency on the average body weight (±SEM) of 2N and Ts65Dn mice during the study. While Ts65Dn mice had significantly lower body weight than the 2N group (p < 0.001), no significant effects of folate deficiency on body weight were detected in either Ts65Dn or 2N mice (p > 0.05). [file Image_1.tiff]
